# Antimicrobial efficacy of Egyptian *Eremina desertorum* and *Helix aspersa* snail mucus with a novel approach to their anti-inflammatory and wound healing potencies

**DOI:** 10.1038/s41598-021-03664-3

**Published:** 2021-12-21

**Authors:** Nessma A. EL-Zawawy, Mahy M. Mona

**Affiliations:** 1 Botany Department, Faculty of Science, Tanta University, Tanta, 31527 Egypt; 2 Zoology Department, Faculty of Science, Tanta University, Tanta, 31527 Egypt

**Keywords:** Drug discovery, Microbiology, Molecular biology, Zoology

## Abstract

Snail mucus is composed of bioactive compounds thought to have different biological properties for the treatment of some skin problems. Although *Helix aspersa* mucus is used in several cosmetic products, a detailed characterization of *Eremina desertorum* mucus composition and its biological activities is still missing. Mucus extracts (MEs) from *H. aspersa and E. desertorum* were prepared and tested for their antimicrobial and anti-inflammatory activities with their potencies in wound healing. Also, chemical characterization was performed by GC–MS analysis. Results showed that ME of *E. desertorum* gave higher inhibitory activity against resistant strains related to burn wound infections compared to ME of *H. aspersa*. Additionally, it revealed a significant anti-inflammatory activity. Moreover, we found that ME of *E. desertorum* lacked cytotoxicity and was able to significantly induce cell proliferation and migration through up-regulation of TGF-β1 and VEGF gene expression. Our results suggested that MEs of *E. desertorum* have higher biological effects than *H. aspersa,* which are attributable to antimicrobial, anti-inflammatory activities, cell proliferation and pave the way for further investigating its potential effect as a human therapeutic agent.

## Introduction

Snails have a thick mucus coating that may aid in minimizing moisture loss, reducing friction, which helps them glide smoothly across dry surfaces, as well as protecting their bodies from physical harm^1^. Mucus secretions have a wide range of functions and biological activity^2^. Trail mucus is mostly composed of large, carbohydrate-rich polymers with a few tiny proteins^3^, which can relieve heartburn as mucus neutralizes stomach acidity and gastroesophageal reflux based on the role of snail mucus in mending ulcers and the role of human mucus in preventing or fighting acidity^4^. Also, snails may produce a large amount of mucin in their mucus secretion, which contains antibacterial proteins and gives them some resistance to infection by pathogens^5^. Moreover, several scientific studies have shown that bioactive compounds-derived from different mucus snails can be utilized in a wide range of therapies, such as creams to treat skin abrasions and scars, respiratory disorders, and heartburn^6^.

Eremina is a very confined genus to many countries of the North African region^7^ and is considered part of the natural ecosystem of Egypt^8^. *Eremina desertorum* is one of the common desert species that occurs in many different locations along the Mediterranean region, between Alexandria till the border of Egypt with Libya^9–11^. Despite the spread of this species in Egypt, to date, there is no study explaining the chemical composition or even proving the medical importance of the mucus extracted from it.

Burn wounds are one of the most important health issues worldwide, especially in the developing countries^12^. Microbial infections for burn wound patients are considered a huge problem, as approximately 50%–75% of mortality in hospitalized burn patients is due to microbial infections^13^. Moreover, the lack of research in Egypt on pathogenicity, resistance of microorganisms from burn wounds and statistical information makes the problem more complicated. Also, many studies on burn wound infections ignored host microbiota-associated pathogens^14^. Recently, Kopeck^15^ reported that the presence of some resistant microbial strains in burns could lower the efficiency of burn wound healing. The wound healing process is controlled by different cytokines and growth factors, such as transforming growth factor-beta 1 (TGF-β1) and vascular endothelial growth factor (VEGF)^16^. TGF-β1 is created by cells such as T cells, platelets and macrophages, which releases neutrophils and fibroblasts to the site of damage at the inflammatory phase of wound healing^17^. Additionally, TGF-β1 helps in the migration, growth, and motivation of fibroblasts^18^. Moreover, VEGF is created by several cells as well as endothelial cells, fibroblasts, platelets and neutrophils^19^. TGF-β1 and VEGF can suppress severe inflammation as inflammation is the response of living tissues to infected wounds. The mechanism of anti-inflammatory agents depends on inhibiting the release of lysosomal constituents of activated neutrophils which can cause tissue damage and inflammation^20^.

Despite the huge commercial diffusion of products from garden snail *Helix aspersa* mucus^21–23^, there have been no reports discussing antimicrobial and anti-inflammatory activities of *E. desertorum* mucus. To our knowledge, there are no studies on the chemical composition of *E. desertorum* mucus related to its biological activities and its mechanisms in wound healing activity. Therefore, the aim of the present study is the first to identify the mucus chemical composition of the desert snail *E. desertorum* compared to the garden snail *H. aspersa* under Egyptian conditions and explore it as a new antimicrobial, and anti-inflammatory approach against resistant pathogens of burn wound infections and its wound healing potency on human skin fibroblasts through the expression of some growth factor genes.

## Results

### Antimicrobial activities and Minimum inhibitory concentrations (MICs)

**Figure 1:**
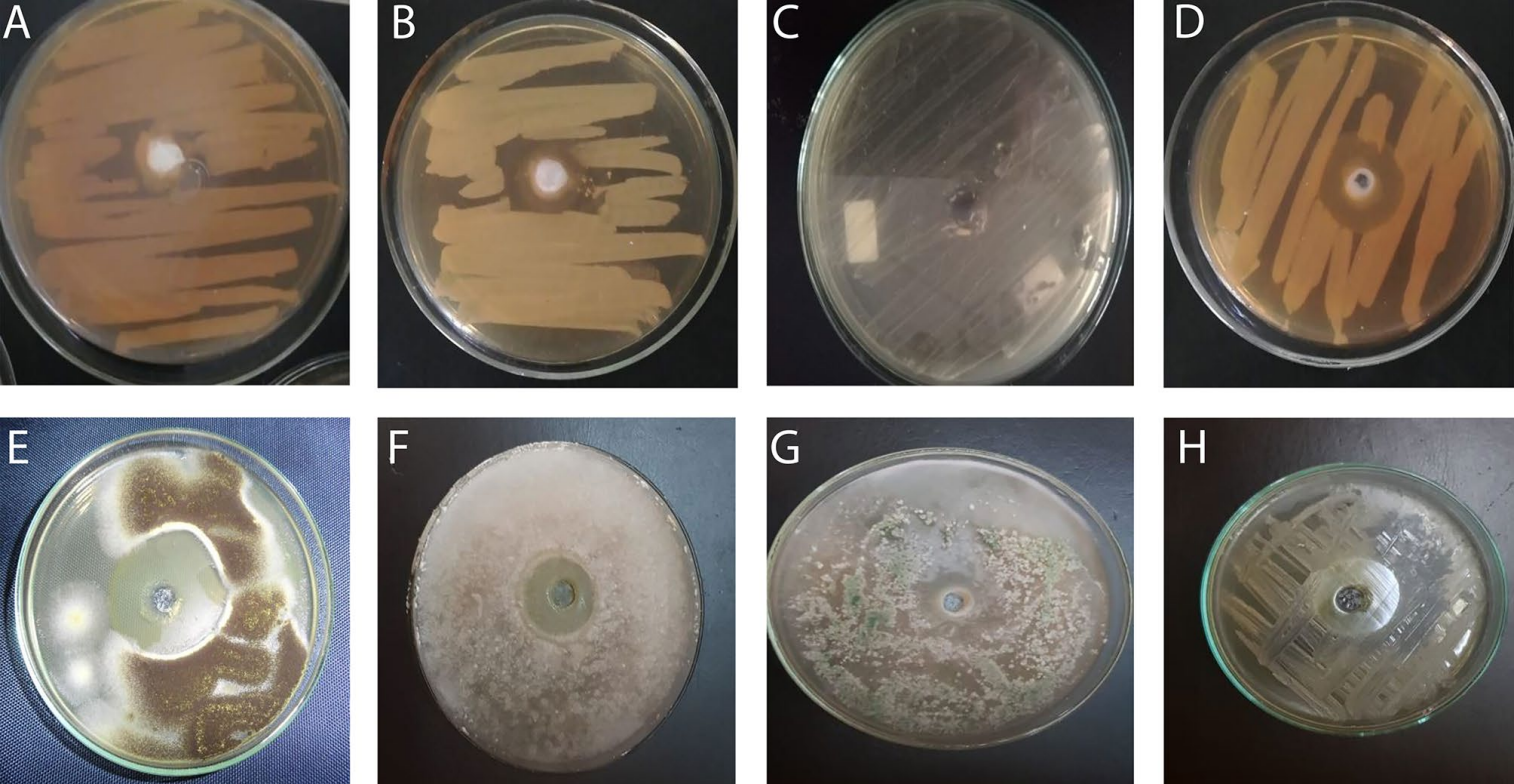
Antimicrobial activities of ME of *E. desertorum* by agar well diffusion method. (**A**) PA-9; (**B**) EC-3; (**C**) KP-1; (**D**) SA-17; (**E**) AN-05; (**F**) RS; (**G**) TH; (**H**) CA-11.

The present study might be the first to investigate effect of MEs of *H. aspersa* and *E. desertorum* against MDR or PDR pathogenic microorganisms isolated from burn wound infections. The antimicrobial activities of both snails were tested against eight resistant pathogens as in Fig. 1. ME of *E. desertorum* showed higher significant inhibitory activity against the tested strains with differences in susceptibility than *H. aspersa*. However, neither snail showed any inhibitory activity against KP-1 (Table 1). Fungal strains were found to be more susceptible strains to MCE of *E. desertorum*. The highest mean zones of inhibition ranged from 3 ± 0.0 to 55.2 ± 0.1 mm and from 9.5 ± 0.0 to 30.5 ± 0.06 mm against fungal and bacterial strains, respectively compared to DMSO (1%) which didn’t show any inhibition zone (Fig. 1). The minimum inhibitory concentrations (MICs) for each organism were shown in Table 1. MIC ranged between (5 and 20 µg/ml) against bacterial strains, while MIC for fungal strains ranged between 7 and 32 µg/ml.

**Table 1:** Antimicrobial activity of MEs of selected snails.

Snails	Microorganisms	Zone of inhibition (mm)					MIC (µg/ml)	DRPs	Resistance type
		Different concentrations (µg/ml)							
		10	20	30	40	50			
**1**	*P. aeruginosa* (PA-9)	0.0 ± 0.0	8 ± 0.1	11.2 ± 0.1	15.5 ± 0.06	15.8 ± 0.06	15	AX, CAZ, FEP, ATM, CRO, IPM, CIP, SXT, C, CN, TOB, K, CT^12^	MDR
**2**		9.5 ± 0.0	13.9 ± 0.1	18.5 ± 0.1	22.8 ± 0.06	25.5 ± 0.06	7		
**1**	*E. coli* (EC-3)	0.0 ± 0.0	7 ± 0.1	10 ± 0.1	15.5 ± 0.06	16.8 ± 0.06	20	AX, CAZ, FEP, ATM, VA, TZP, AMC, IPM, CIP, SXT, C, TOB, K, CT^20^	MDR
**2**		10.5 ± 0.0	14.5 ± 0.1	20.5 ± 0.1	25.6 ± 0.06	30.5 ± 0.06	5		
**1**	*K. pneumonia* (KP-1)	0.0 ± 0.0	0.0 ± 0.0	0.0 ± 0.0	0.0 ± 0.0	0.0 ± 0.0	ND	PIP, AMC, TZP, CTX, NA, AK, VA, ATM, C, CIP, CN, CRO, CTX, IPM, K, SXT, TE^20^	PDR
**2**		0.0 ± 0.0	0.0 ± 0.0	0.0 ± 0.0	0.0 ± 0.0	0.0 ± 0.0	ND		
**1**	*S. aureus* (SA-17)	0.0 ± 0.0	8.5 ± 0.06	10 ± 0.06	14.9 ± 0.1	18.2 ± 0.1	15	PIP, AMC, TZP, CTX, CFP, NA, AK, AX, C, CIP, CN, CTX, FEP, IPM, K, SXT, TE^25^	PDR
**2**		10 ± 0.0	16.5 ± 0.1	19 ± 0.1	21.8 ± 0.06	28 ± 0.06	5		
**1**	*A. niger* (AN-05)	0.0 ± 0.0	0.0 ± 0.0	0.0 ± 0.0	6.2 ± 0.06	8 ± 0.1	32	AMB, ITC, CLT, MIZ, FLC, TRB^25^	MDR
**2**		10.8 ± 0.0	20.8 ± 0.0	34.6 ± 0.06	47.2 ± 0.06	55.2 ± 0.1	7		
**1**	*R. stolonifer* (RS)	0.0 ± 0.0	0.0 ± 0.0	9.2 ± 0.0	12.6 ± 0.0	14 ± 0.1	25	AMB, ITC, CLT, MIZ, FLC, NYT^26^	MDR
**2**		8.5 ± 0.1	10.8 ± 0.1	27.5 ± 0.1	40.8 ± 0.0	52.6 ± 0.0	10		
**1**	*Trichoderma harzianum* (TH)	0.0 ± 0.0	0.0 ± 0.0	10 ± 0.0	14.6 ± 0.0	18 ± 0.1	25	AMB, ITC, CLT, MIZ, FLC, MCFG, NYT^26^	MDR
**2**		8 ± 0.1	10.2 ± 0.1	25.5 ± 0.1	38.8 ± 0.0	49.6 ± 0.0	10		
**1**	*C. albicans* (CA-11)	0.0 ± 0.0	3 ± 0.0	8.6 ± 0.06	10.8 ± 0.1	13 ± 0.06	20	AMB, ITC, CLT, MIZ, FLC, MCFG, NYT, TRB^26^	PDR
**2**		7.8 ± 0.0	15.2 ± 0.1	26.8 ± 0.1	38.6 ± 0.1	49.2 ± 0.06	12		
ANOVA	*p* value	< 0.001	< 0. 001	< 0. 001	< 0. 001	< 0. 001	–	–	–
	F	34.15	64,095	154.4	73,302	36,211	–	–	–

Values are the mean of three replicates ± SD. *P* value < 0.05 is considered significant.

Data obtained from our previous studies^12,20,25,26^.

**1**: *H.aspersa*; **2**: *E. desertorum*; ND, Not detected; MIC, Minimum inhibitory concentration; DRPs, Drug-resistance patterns; MDR, Multi-drug resistance; PDR, Pan-drug resistance; AX, Amoxicillin; CAZ, Ceftazidime; FEP, Cefepime; ATM, Aztreonam, CRO, Ceftriaxone; IMP, Imipenem; CIP, Ciprofloxacin; SXT, Cotrimoxazole; C, Chloramphenicol; CN, Gentamicin; TOB, Tobramycin; K, Kanamycin; CT, Colistin sulfate; PIP, Piperacillin; AMC, Amoxicillin/clavulanic acid; TZP, Pipracillin/tazobactam; CTX, Cefotaxime; CFP, Cefoperazone; NA, Nalidixic acid; AK, Amikacin; TE, Tetracycline; VA, Vancomycin; AMB, Amphotericin; FLC, Fluconazole; ITC, Itraconazole; CLT, Clotrimazole; MIZ, Miconazole; MCFG, Micfungin; NYT, Nystatin; TRB, Terbinafine.

### Anti-inflammatory activities of MEs of *H. aspersa* and *E. desertorum*

**Figure 2:**
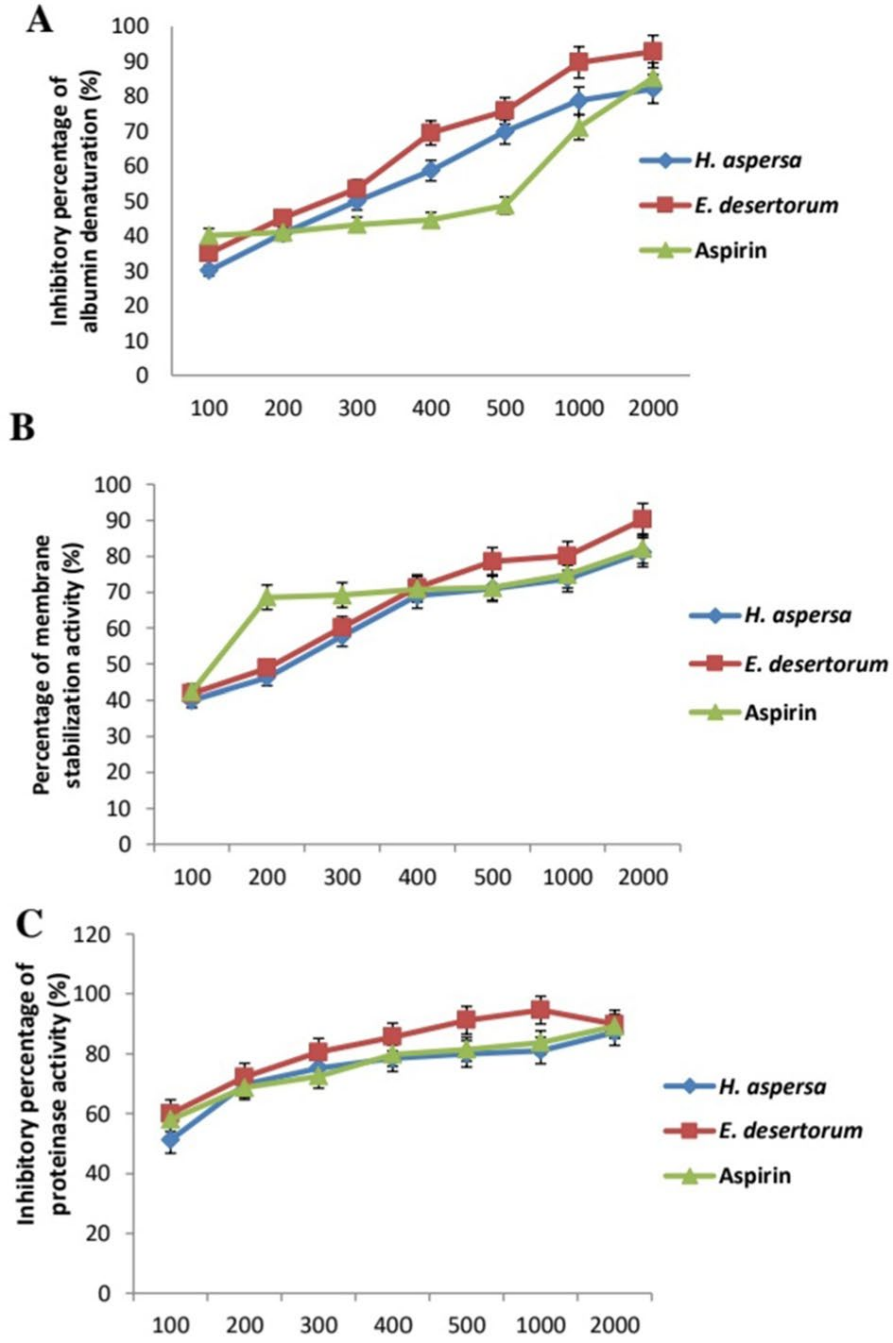
Anti-inflammatory activities of MEs of both snails compared to aspirin. Results represent the average of three independent experiments ± SD. *p < 0.05.

The anti-inflammatory activities of MEs of both snails were determined through membrane stabilization, albumin denaturation, and proteinase inhibitory activity compared with aspirin as a reference drug (Fig. 2). Both snails showed anti-inflammatory activities, while *E. desertorum* showed higher activity. *E. desertorum* showed highly significant stabilization toward the human red blood cell membrane. Also, the percentage inhibition of albumin denaturation for *E. desertorum* at a concentration of 2000 µg/ml was higher than that of aspirin at the same concentration, with inhibition rates of 92.8% and 85.3%, respectively. Moreover, a significant increase in proteinase activity inhibition was highly similar to that of aspirin with inhibition of 89.9% and 89.2%, respectively at a concentration of 2000 µg/ml.

### Lack of cytotoxicity of MEs of *H. aspersa* and *E. desertorum*

**Figure 3:**
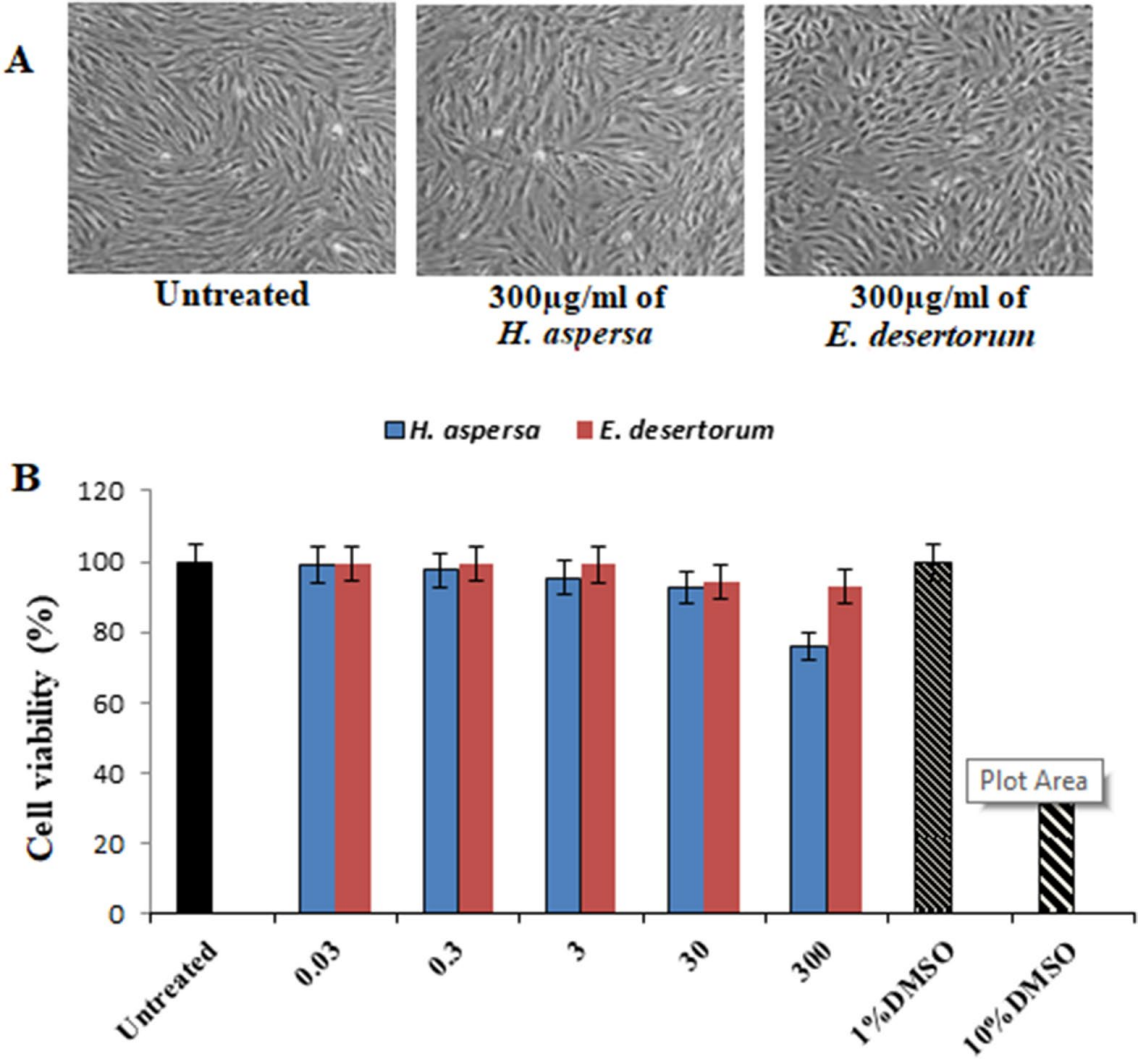
Cytotoxicity evaluation of MEs of both selected snails. HSF cells were exposed to 300 µg/ml of MEs of both selected snails and cell viability was examined by SRB assay. (**A**) Representative images with magnification of (10 ×) taken by light microscopy of HSF cells untreated and treated with 300 µg/ml of both selected strains at 48 h. (**B**) Cell viability was calculated at 24, 48 and 72 h compared to untreated cells (control), DMSO (10%) and (1%) were used as positive and vehicle controls of cell death, respectively. Results represent the average of three independent experiments ± SD. *p < 0.05.

To evaluate the biological effects of MEs of both snails, human skin fibroblast (HSF) cells were treated in vitro with different concentrations (0.03–300 µg/ml) of both snails, to show their effect on normal cell viability and morphology. Figure 3A,B showed lack of cytotoxicity of both snails as the percentage viability of HSF cells at the highest treated concentration of MEs of *H. aspersa* and *E. desertorum* was observed to be 93% and 75.8%, respectively compared to untreated samples and DMSO (1%) and (10%) as different controls. The concentrations of MEs of both snails used for treatment and their corresponding percentage cell viability showed IC_50_ > 300 µg/ml in both snails which confirmed the disappearance of any toxic effect of treated concentration.

### Induction of MEs of both snails to cell migration and wound repair

**Figure 4:**
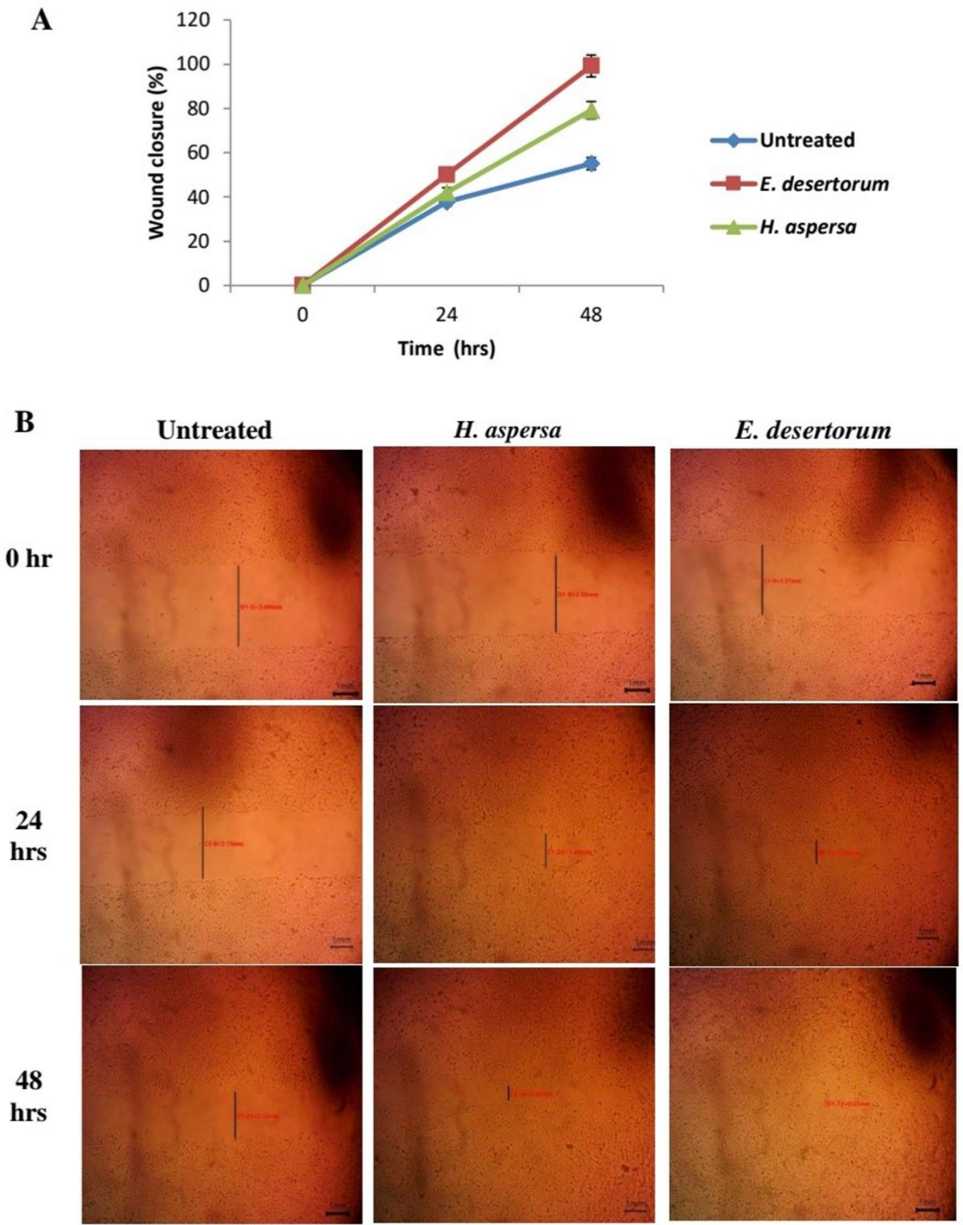
Scratch-wound healing assay. (**A**) Percentage of wound closure at 0, 24, 48 h in the absence and presence of MEs of both selected snails (300 µg/ml). Results represent the average of three independent experiments ± SD. *p < 0.05. (**B**) Microscopical representative images for wound healing of MEs of both selected snails.

In addition to the cell viability, the cell migration and proliferation properties of both snails were determined by the scratch wound assay. As shown in Fig. 4A, both snails improved the wound healing process compared to untreated cells as ME of *E. desertorum* induced the migration of HSF cells resulting in complete wound closure after 48 h. faster than ME of *H. aspersa.* Figure 4B indicated that at 300 μg/ml, *E. desertorum* closed the gap created by the scratch by 99.2% after 48 h. While in untreated cells, 55.1% of the gap was closed at 48 h.

### Upregulation of TGF-β1 and VEGF genes expression

**Figure 5:**
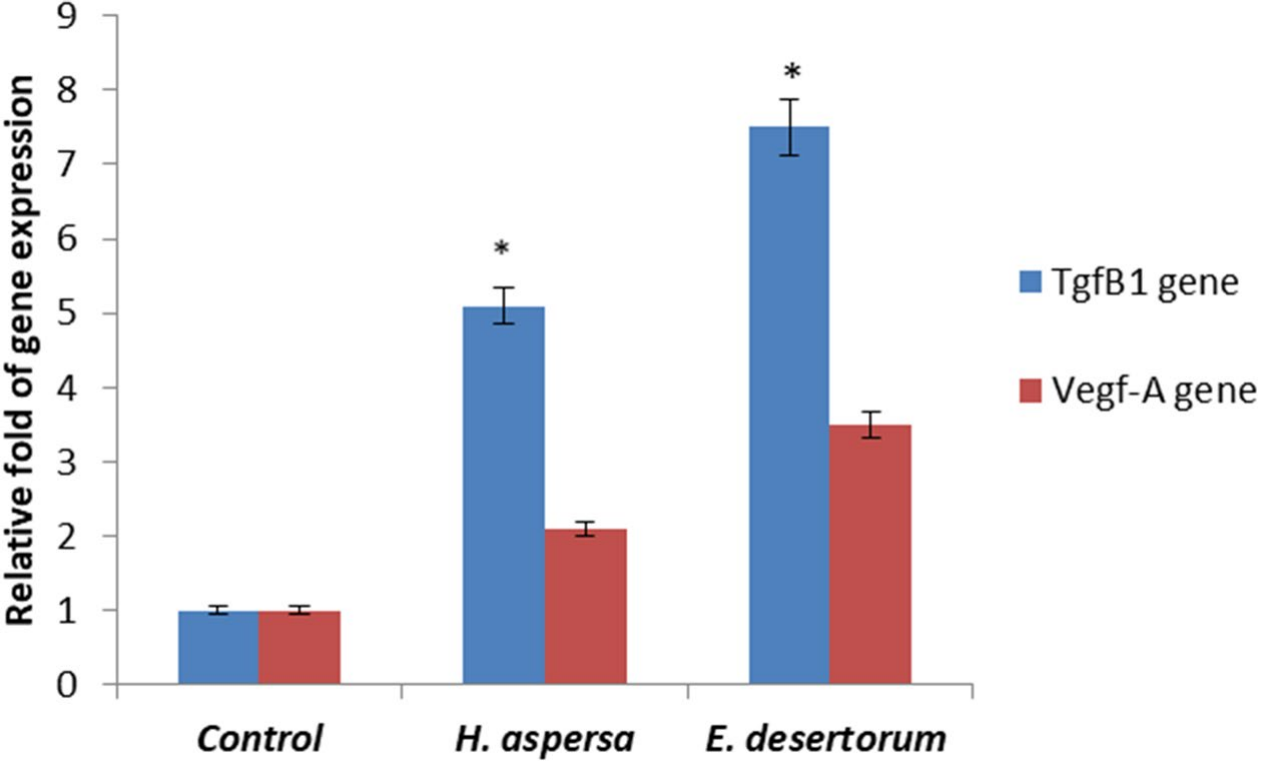
Effect of MEs of both snails (300 µg/ml) on the expression of Tgfβ1 and Vegf-A genes in HSF cells after 48 h. Results represent the average of three independent experiments ± SD. *p < 0.05.

The present investigation determined changes in the expression of TGF-β1 and VEGF genes by real-time-PCR in HSF cells with MEs of both snails at 48 h after treatment. To determine the possible molecular mechanism of the induction of MEs of both snails to wound repair and healing, we tested the expression levels of TGF-β1 and VEGF genes. Expression of TGF-β1 gene treated by MEs of *H. aspersa* and *E. desertorum* was significantly upregulated by fivefold, and 7.5-fold, respectively, when compared to the control (Fig. 5). Also, expression of VEGF gene was significantly upregulated by two fold, and 3.5-fold when treated with MEs of *H. aspersa* and *E. desertorum*, respectively.

### Chemical analysis of MEs of both snails using GC–MS

Chemical constituents, molecular weight and peak area of each component for MEs of both snails were listed in Tables 2 and 3. Our results indicated that the major compounds in the ME of *E. desertorum* were 3H-1,2,4-triazole-3-thione, 4,5-dihydro-4,5-diphenyl followed by phthalic acid, 7-bromoheptyl ethyl ester and methyl 1,2-benzisothiazole-3-acetate. In the ME of *H. aspersa,* the major compounds were thiophene, 3-(decyloxy)tetrahydro-, 1,1-dioxide followed by 4-(nonafluoro-tert-butyl) nitrobenzene. Therefore, further study will be performed for the isolation and purification of these active compounds with a comprehensive toxicological analysis to determine their safety as it is beyond the scope of this paper.

**Ta﻿ble 2:** Chemical constituents ME of *E. desertorum* using GC–MS.

Peak	Name of compound	Molecular formula	Molecular weight (g/mol)	Retention time (min)	Peak area (%)
1	Bis(trimethylsiloxy)methylsilane	C_7_H_21_O_2_Si_3_	221.50	5.376	1.11
2	4-Trimethylsilyl-9,9-dimethyl-9-silafluorene	C_17_H_22_Si_2_	282.5	5.620	1.72
3	3,6,9,12,15,18-Hexaoxanonadecan-1-ol, TMS derivative	C_16_H_36_O_7_Si	368.54	5.829	0.57
4	Decamethyl cyclo penta siloxane	C_10_H_30_O_5_Si_5_	370.77	5.984	2.47
**5**	Methyl 1,2-benzisothiazole-3-acetate	C_10_H_9_NO_2_S	207.25	7.507	5.39
6	4-(Nonafluoro-tert-butyl) nitrobenzene	C_10_H_4_F_9_NO_2_	341.13	7.613	2.07
7	Benzeneacetaldehyde, .alpha.-(methoxymethylene)-4-nitro-	C_10_H_9_NO_4_	207.18	7.937	1.54
8	Ehoxytris(trimethylsiloxy)silane	C_11_H_32_O_4_Si_4_	340.71	7.978	0.63
9	1-(3,6,6-Trimethyl-1,6,7,7a-tetrahydrocyclopenta[c]pyran-1-yl)ethanone	C_13_H_18_O_2_	206.28	8.025	1.73
10	5H-Dibenzo[a,d]cyclohepten-5-amine	C_15_H_13_N	207.27	8.055	2.05
11	Methyl 1,2-benzisothiazole-3-acetate	C_10_H_9_NO_2_S	207.25	8.989	1.23
12	Methyl 2-oxo-1,2,5,6,7,8-hexahydroquinoline-3-carboxylate	C_11_H_13_NO_3_	207.23	9.084	1.97
**13**	Phthalic acid, 7-bromoheptyl ethyl ester	C_17_H_23_BrO_4_	371.3	9.148	7.29
14	Diethyl Phthalate	C_6_H_4_(COOC_2_H_5_)_2_	222.24	9.250	3.26
15	Prop-2-enoic acid, 2-cyano-3-(3-methyl-2-thienyl)-, methyl ester	C_10_H_9_NO_2_S	207.25	9.302	1.44
16	Isophthalic acid, 2-methoxyethyl isobutyl ester	C_15_H_20_O_5_	280.32	9.369	1.64
17	Prop-2-enoic acid, 2-cyano-3-(3-methyl-2-thienyl)-, methyl ester	C_10_H_9_NO_2_S	207.25	9.467	1.24
18	7,7,9,9,11,11-Hexamethyl-3,6,8,10,12,15-hexaoxa-7,9,11-trisilaheptadecane	C_14_H_36_O_6_Si_3_	384.69	9.904	2.72
19	Silicic acid, diethyl bis(trimethylsilyl) ester	C_10_H_28_O_4_Si_3_	296.58	10.004	1.63
20	Benzene, [1-(3-butenylthio)-2-nitroethyl]-	C_12_H_15_NO_2_S	237.32	10.506	2.31
21	Benzothiophene-3-carboxylic acid,4,5,6,7-tetrahydro-2-amino-6-ethyl-, ethyl ester	C_13_H_19_NO_2_S	253.36	11.107	3.58
22	Propanephosphonic acid, bis(trimethylsilyl) ester	C_9_H_25_O_3_PSi_2_	268.44	11.291	3.68
**23**	3H-1,2,4-triazole-3-thione, 4,5-dihydro-4,5-diphenyl-	C_14_H_11_N_3_S	253.32	11.483	6.07
24	Cyclotrisiloxane, hexamethyl-	C_6_H_18_O_3_Si_3_	222.46	11.686	0.95
25	1,2-Bis(trimethylsilyl)benzene	C_12_H_22_Si_2_	222.47	11.712	0.34
26	6-Methyl-2-(3-nitrophenyl)imidazo[1,2 a]pyridine	C_14_H_11_N_3_O_2_	253.26	12.052	1.83
27	Phthalic acid, 7-bromoheptyl ethyl ester	C_17_H_23_BrO_4_	371.3	12.130	2.84
28	Diethyl Phthalate	C_12_H_14_O_4_	222.24	12.163	4.26
29	Benzothiophene-3-carboxylic acid, 4,5,6,7-tetrahydro-2-amino-6-ethyl -, ethyl ester	C_13_H_19_NO_2_S	353.36	12.267	4.12
30	6Methyl2(3nitrophenyl)imidazo[1,2-a]pyridine	C_14_H_11_N_3_O_2_	253.26	12.561	1.13
31	Methyl 6,6,8,8,10,10-hexamethyl-3-oxo-2,5,7,9,11-pentaoxa-6,8,10-trisilatridecan-13-oate	C_12_H_28_O_8_Si_3_	348.60	12.693	1.58
32	1,1,1,3,5,5,5-Heptamethyltrisiloxane	C_7_H_21_O_2_Si_3_	221.50	13.830	0.45
33	Cyclohexa-2,5-diene-1,4-dione, 2-methyl-5-(4-morpholinyl)-	C_11_H_13_NO_3_	207.23	13.848	0.19
34	Methyltris(trimethylsiloxy)silane	C_10_H_30_O_3_Si_4_	310.68	14.040	0.75
35	9H-Fluorene-4-carboxylic acid, 9-oxo-, (2,6-dimethylphenyl)amide	C_22_H_17_NO_2_	327.4	14.270	0.84
36	Benzothiophene-3-carboxylic acid, 4,5,6,7-tetrahydro -2-amino-6-ethyl -, ethyl ester	C_13_H_19_NO_2_S	353.36	15.820	2.10

**Table 3:** Chemical Constituents ME of *H. aspersa* using GC–MS.

Peak	Name of compound	Molecular formula	Molecular weight (g/mol)	Retention time (min)	Peak area (%)
1	(Z)-2-Heptene	C_7_H_14_	98.1861	5.163	1.11
2	4H-Thiopyran-4-one, tetrahydro-, 1,1-dioxide	C_5_H_8_O_3_S	148.18	5.186	2.12
**3**	Thiophene, 3-(decyloxy)tetrahydro-, 1,1-dioxide	C_14_H_28_O_3_S	276.44	5.245	4.85
**4**	Thiophene, 3-(decyloxy)tetrahydro-, 1,1-dioxide	C_14_H_28_O_3_S	276.44	5.300	6.12
5	2-Ethylacridine	C_15_H_13_N	207.27	5.369	1.73
6	Auramine	C_17_H_21_N_3_	267.37	5.621	2.18
7	Methyltris(trimethylsiloxy)silane	C_10_H_30_O_3_Si_4_	310.68	5.854	3.00
8	N-(Trifluoroacetyl)-N,O,O',O''-tet rakis(trimethylsilyl)norepinephrin	C_22_H_42_F_3_NO_4_Si_4_	553.9	5.985	3.07
**9**	4-(Nonafluoro-tert-butyl) nitrobenzene	C_10_H_4_F_9_NO_2_	341.13	7.526	4.86
10	Cyclohexasiloxane, dodecamethyl-	C_12_H_36_O_6_Si_6_	444.92	7.614	3.57
11	3-Isopropoxy-1,1,1,5,5,5-hexamethy l-3-(trimethylsiloxy)trisiloxane	C_12_H_34_O_4_Si_4_	354.74	8.857	4.14
12	Mercaptoethanol, 2TMS derivative	C_8_H_22_OSSi_2_	222.50	9.916	1.95
13	Octasiloxane, 1,1,3,3,5,5,7,7,9,9,11,11,13,13,15,15-hexadecamethyl-	C_16_H_48_O_7_Si_8_	577.2	10.011	2.13
14	N-(2-Acetylcyclopentylidene)cyclohexylamine	C13H21NO	207.31	10.507	2.16
15	6-Chloro-4-phenyl-2-propylquinolin	C_18_H_16_ClN	281.8	5.163	1.11

## Discussion

Based on previous investigations, antimicrobial activities of mucus from mollusks including snails and slugs have never been suggested extensively^24^. According to several reports, antimicrobial activity depends on snail species, extraction method, and the resistance of the tested organism^25^. In the present study, ME of *E. desertorum* was the most effective snail against the most selected resistant strains with a strong inhibitory activity. These results were similar to those Lopez^26^ who evaluated the antimicrobial activity of the crude extract of the marine snail *C. muricatus*. Although, there are few reports on the potent antimicrobial activities of extracts from *H. aspersa*, our study is considered the first to explore the antimicrobial activities of *E. desertorum* compared to *H. aspersa* against resistant pathogens related to burn wound infection.

The ME of *E. desertorum* showed significant anti-inflammatory activity through membrane stabilization, albumin denaturation, and proteinase inhibitory activity compared with commercial aspirin. This might be the first study to discuss the in vitro anti-inflammatory activity of this snail. Therefore, we suggest it as a new alternative agent with a potent anti-inflammatory activity in the treatment of burn wound infections. Hence, ME of *E. desertorum* treatment was further conducted to evaluate the efficacy of this snail in curing burn wound infections.

Moreover, ME of *E. desertorum* accelerates wound healing by inducing the migration of fibroblasts and enhancing the expression of wound healing related genes (TGF-β1 and VEGF). This is in agreement with Coppe^27^ who demonstrated that the methanolic extract of *C. molmol* and the ethanolic extract of henna significantly improved the expression of TGF-β1 and VEGF genes at 48 h. after treatment of normal mouse fibroblast cells. However, there are some reports on wound healing activity of mucus of different snails^28^, there are no reports on effect of this snail on expression of wound healing related genes.

It was necessary as a next step to check the chemical composition of bioactive compounds in both snails. The differences in their biological activities may be due to differences in the active compounds present in both snails. GC–MS analysis indicated that the chemical constituents of the most promising ME of *E. desertorum snail* had 3 major different peaks compared to *H. aspersa*; which are 3H-1,2,4-triazole-3-thione,4,5-dihydro-4,5-diphenyl followed by phthalic acid, 7-bromoheptyl ethyl ester and methyl 1,2-benzisothiazole-3-acetate. Similarly, the first compound showed a potent antibacterial activity^29^. The second component was reported to have several biological activities^30^. In addition, the third one showed a strong antimicrobial activity^31^. While in Egyptian *H. aspersa*, there were another two major compounds; thiophene, 3-(decyloxy)tetrahydro-, 1,1-dioxide followed by 4-(nonafluoro-tert-butyl) nitrobenzene which had different biological activities^32,33^. This variation in mucus composition could be attributed to species differences, as well as mechanical factors such as temperature, humidity, light intensity, soil conditions, and food supply. These data agreements with Meikle^34^ who found substantial differences between the mucus of six coral species. Also, Sallam^35^ observed several chemical variations in the composition of three common Egyptian land snails, *Eobania vermiculata*, *Theba pisana* and *Monacha obstructa* mucus. Between the two species in this study, it should not be surprising that different forms of mucus have different compositions and different mechanical properties according to their environmental living conditions. These environmental conditions also affect the physical properties of the two snail species in terms of color and viscosity. The garden snail *Helix aspersa* was colorless and less viscous compared with mucus dessert snail *Eremina desertorum* which was slightly cloudy-white with high viscous. Dessert snails with high viscosity acted as barriers, preventing moisture loss and safeguarding snails from bacterial infection^1,36^. Finally, these results suggest that *E. desertorum* snail is a mixture of several compounds, and each component might contribute to its biological activity more than if they acted alone. Therefore, the current study suggested that ME of *E. desertorum* snails is a potential source of natural components that possess antimicrobial and anti-inflammatory properties that may be used for the treatment of burn wound infections. Also, it can induce wound healing by improving the expression of growth factors genes. However, to date, there are no available toxicological data on human regarding the *E. desertorum* snails; therefore, further assessment should be performed to define the safe doses of this novel snail for human use.

## Conclusion

This study has evidenced the efficacy of ME of *E. desertorum* snail as a new antimicrobial and anti-inflammatory agent in burn wound infections, highlighting its efficiency in wound healing for future usage in topical technology. Moreover, in vivo and human studies need to be performed further to confirm the biological properties of snails.

## Material and methods

### Snail collection and mucus extraction

Thirty adult garden snail *Helix aspersa* and desert snail *Eremina desertorum* were collected from Foah region, Kafr El-sheikh, Egypt (31° 06′ 42″ N 30° 56′ 45″ E) and El Alamein, Western Coast, Egypt (30° 50′ N 28° 57′ E), respectively. The samples were identified according to Schileyko^37^ as reported in the supplementary data (Fig. S1). Each species of snail was housed in two separate plastic boxes, each with 15 snails. To keep the plastic boxes damp, they were sprayed with water every day. Then snails were transferred individually packaged in plastic containers and stored. To avoid infection, leave snails 3 days without eating.

Snails were manually stimulated at the pedal glands in their foot. Each individual's mucus sample was collected and then pooled for each species. About 100 ml of crude extract from 25 snails of each species was collected. The harvested mucus was filtered. Mucus was then sterilized by filtering through 0.45-µm membrane and stored at − 80 °C. To obtain only the dry part, mucus samples were lyophilized overnight to obtain a solid powder that was used for biological characterization.

### Microbiological characterization

Bacterial contamination was tested by plating 100 µl of mucus extracts (MEs) of both snails on tryptic Soy agar (TSA) medium (Biomerieux, Italy). Colonies were counted after incubation for 24–48 h. at 37 °C and expressed as colony forming unit (CFU). Also, fungal and yeast contamination was evaluated by plating 100 µl of MEs of both specimens on Sabouraud medium plates (Biomerieux, Italy). Fungal growth was noticed after incubation for 5–7 days at 30°C^23^. Microbiological evaluation of MEs of selected strains is reported in supplementary data (Table S1), which confirmed the sterilization of MEs of both snails by the absence of fungal and bacterial contaminations without the addition of any preservative.

### Microbial strains

Eight clinically resistant bacterial and fungal strains used in this study were isolated previously from burn wound infections. The pathogenic bacteria were *P. aeruginosa* (PA-9)^12^, *E. coli* (EC-3)^20^, *K. pneumonia* (KP-1) and *S. aureus* (SA-17)^20,38^. While, pathogenic fungal strains were *A. niger* (AN-05), *R. stolonifer* (RS), *Trichoderma harzianum* (TH) and *C. albicans* (CA-11)^38,39^. All isolates were identified as MDR or PDR strains as described previously in our studies^12,38,39^ and stored at − 70 °C. Active cultures for further experiments were prepared by transferring a loop full of culture from frozen glycerol stock cultures of each strain to test tubes of Mueller–Hinton broth (MHB) (Merck, Darmstadt, Germany) for bacteria, and Sabouraud Dextrose (SD) broth for fungi, and were incubated for 24–48 h at 37 °C.

### Antimicrobial activity assay

Antimicrobial activities of MEs from both snails were assessed against the eight selected strains by the agar well diffusion method^40^. The agar plates were swabbed with 100 µl of each selected strains (1 × 10^6^ cells/ml). Wells were made in agar plates using a sterile cork borer of 5 mm. MEs were dissolved in 1% pure dimethyl sulfoxide (DMSO; Sigma-Aldrich, St. Louis, Missouri, USA) to a final concentration of 100 µg/ml. Twenty microliters of various concentrations (10, 20, 30, 40, 50 µg/ml) were added to each well. DMSO (1%) was used as a negative control. Then, these plates were incubated at 37 °C for 48 h. After the incubation period, the results were observed and the diameter of the inhibition zone around each well was measured. All tests were performed in triplicate.

### Determination of minimum inhibitory concentrations (MICs)

Minimal inhibitory concentration (MICs) of MEs from both snails against the eight selected strains was determined by microdilution method^41^. The growth was observed and the optical density was read at 595 nm spectrophotometrically. MIC of each extract was determined by the lowest concentration of sample that inhibited the development of turbidity.

### Anti-inflammatory activity

The anti-inflammatory activities of MEs from both snails were determined in vitro by three experiments as described in our previous studies^12,42^ in details; membrane stabilization of human red blood cells, albumin denaturation and proteinase inhibitory activity. Different concentrations (100, 200, 300, 400, 500, 1000 and 2000 μg/ml) of both snails and aspirin (Bayer, Leverkusen, Germany) as a reference drug were prepared and compared with DMSO (1%) as a negative control.

### Cell culture

Human Skin Fibroblast (HSF) cell line employed in this study was obtained from Nawah Scientific Inc., (Mokatam, Cairo, Egypt). Cells were maintained in DMEM media supplemented with 100 mg/ml of streptomycin, 100 units/ml of penicillin and 10% of heat-inactivated fetal bovine serum in humidified, 5% (v/v) CO_2_ atmosphere at 37 °C. Cells were counted by a hemocytometer and viability was calculated to seed the cells at appropriate densities, to perform the assays.

### Cell viability and cytotoxicity studies

The cytotoxicity of MEs of both snails on HSF cells was evaluated by SRB assay^43^. Briefly, HSF cells with initial density (5 × 10^3^ cells) were seeded in 96-well plates and incubated with 100 μl of DMEM media for 24 h. Cells were then treated with another aliquot of 100 μl media containing MEs of both snails separately at various concentrations (0.03, 0.3, 3, 30, 300 μg/ml). After 72 h of treatment exposure, cells were fixed by replacing media with 150 μl of 10% TCA and incubated at 4 °C for 1 h. The TCA solution was removed, and the cells were washed 5 times with distilled water. Aliquots of 70 μl SRB solution (0.4% w/v) were added and incubated in a dark place at room temperature for 10 min. Plates were washed 3 times with 1% acetic acid and allowed to air-dry overnight. Then, 150 μl of Tris (10 mM) was added to dissolve protein-bound SRB stain; the absorbance was measured at 540 nm using a BMG LABTECH®- FLUOstar Omega microplate reader (Ortenberg, Germany). The cells treated with DMEM alone, 1% DMSO and 10% DMSO were considered as negative, vehicle and positive controls, respectively^23^.

### Scratch assay and assessment of cell migration

The wound healing properties of MEs of both snails were tested on HSF cells scratch assay^44^. Briefly, cells were seeded at density of 3 × 10^5^ cells/well in 6-well plate and were cultured overnight. After 24 h. medium was removed and a linear scratch in the middle of the well was done using a p200 tip. Then, 400 μl of selected snails with a concentration of 300 µg/ml or media (control) were added to each well. Scratch repair and cell migration were observed in the images taken by an inverted microscope, equipped with a digital camera. The experiments were performed in triplicate. The width of the scratch and wound closure at different time intervals (0, 24 and 48 h.) was analyzed by MII Image View software version 3.7.

### Real time PCR (qRT-PCR) expression analysis

Effect of MEs of selected snails on the expression of transforming growth factor-beta 1 gene TGF-β1 and vascular endothelial growth factor gene (VEGF), was evaluated by qRT-PCR. Hot phenol/chloroform extraction method^45^ was used in extraction of total RNA. The obtained cDNA was then used for real-time polymerase chain reaction (PCR) using master SYBR Green I (Takara Bio, Japan) on ABI 7900HT. Real-time PCR was executed at 95 °C for 10 s, 62 °C for 15 s, and 72 °C for 8 s using the primers for normalizing GAPDH gene against the Tgfβ1 and Vegf-A target genes. Primers were designed by GenScript according to the cDNA sequences of mouse TGF-β1 and VEGF and GAPDH in GeneBank as shown in S2. Table 2. Real-time PCR was performed in triplicate for every cDNA. Expression in fibroblast cells treated with each extract at 24 and 48 h was compared with the control (non-treated cells) after normalization with GAPDH. We used relative gene expression, to identify the increase or decrease of a transcript of target gene in treated sample versus control sample by normalizing with a housekeeping gene. To determine the difference in gene expression between groups, the data were analyzed using the Relative Expression Software Tool (REST; version 2009).

### Gas chromatography-mass spectrometer (GC–MS) analysis

MCEs of both snails were investigated for their phytoconstituents using GC–MS (Trace GC Ultra, USA), at the National Research Centre (NRC), El Dokky, Giza Governorate. Identification of unknown compounds was based on comparing their retention time relative to those of the known compounds by matching spectral peaks available with Wiley 9 Mass Spectral Library^46^.

### Statistical analysis

All data were expressed as mean ± standard deviation of three replicates and submitted to variance analysis (ANOVA) using SPSS-20. Statistical differences were considered to be significant at *p < 0.05.

## Supplementary Information

Supplementary Information.

## Data Availability

The datasets used and analyzed during this study are available from the corresponding author upon request.
