# Supplementary material for: Antimicrobial efficacy of Egyptian Eremina desertorum and Helix aspersa snail mucus with a novel approach to their anti-inflammatory and wound healing potencies
Source: Sci Rep. 2021 Dec 21;11:24317. doi: 10.1038/s41598-021-03664-3 (PMC8692597; doi:10.1038/s41598-021-03664-3)
Supplement: Supplementary file 1 — Supplementary Information. [file 41598_2021_3664_MOESM1_ESM.docx]

**Supplementary Data**

**Table S1: Microbiological analysis of MEs of selected snails**

| **Sample 1** | | |
| --- | --- | --- |
| **Specification** | **Values** | **Measure unit** |
| Gram + | 0 | CFU |
| Gram - | 0 | CFU |
| Fungi | 0 | CFU |
| Yeast | 0 | CFU |
| **Sample 2** | | |
| Gram + | 0 | CFU |
| Gram - | 0 | CFU |
| Fungi | 0 | CFU |
| Yeast | 0 | CFU |

Sample1: *H. aspersa*; Sample 2: *E. desertorum*; CFU: Colony forming unit

**Table S2: Sequence of designed primers for Tgfβ1 and Vegf-A genes**

| **Gene** | **Forward primer (5′-3′)** | **Reverse primer (5′-3′** |
| --- | --- | --- |
| GAPDH | ATGACTCTACCCACGGCAAG | CTGGAAGATGGTGATGGGTT |
| Tgfβ1 | CTGAACCAAGGAGACGGAAT | GGTTCATGTCATGGATGGTG |
| Vegf-A | GTCCTCACTTGGATCCCGACA | CCTGGCAGGCAAACAGACTTC |


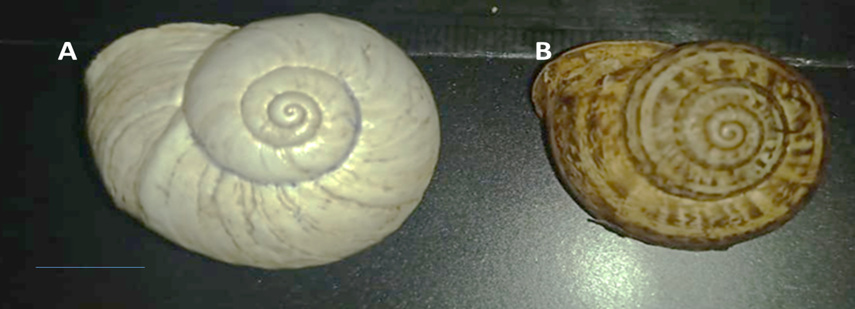


**Fig (S1): Shells of both collected snails**

**(A)** Desert snail *Eremina desertorum* (Forsskål, 1775): Helicidae (family), Helicinae Rafinesque, 1815 (subfamily), Gastropoda Pulmonata (class), Mollusca (phylum).

**(B)** Garden snail *Helix aspersa* (Müller, 1774): Helicidae (family), Helicinae Rafinesque, 1815 (subfamily), Gastropoda Pulmonata (class), Mollusca (phylum).
